# A theory of change roadmap for universal health coverage in India

**DOI:** 10.3389/fpubh.2022.1040913

**Published:** 2022-12-01

**Authors:** Angela Chaudhuri, Nilakshi Biswas, Shiv Kumar, Asha Jyothi, Ranjani Gopinath, Nachiket Mor, Preethi John, Thelma Narayan, Mirai Chatterjee, Vikram Patel

**Affiliations:** ^1^Catalyst Group, Swasti Health Catalyst, Bangalore, India; ^2^The Banyan Academy of Leadership in Mental Health, Thiruvidanthai, India; ^3^Global Business School for Health, University College London, London, United Kingdom; ^4^Centre for Public Health and Equity (SOCHARA), Bangalore, India; ^5^Self-Employed Women's Association (SEWA), Ahmedabad, India; ^6^Department of Global Health and Social Medicine, Harvard Medical School, Boston, MA, United States; ^7^Department of Global Health and Population, Harvard TH Chan School of Public Health, Boston, MA, United States

**Keywords:** Universal Healthcare, Theory of Change, Citizens' Commission, UHC roadmap, nested Theory of Change

## Abstract

The Theory of Change (ToC) approach is one of the methodologies that the Lancet Citizens' Commission has chosen to build a roadmap to achieving Universal Healthcare (UHC) in India in the next 10 years. The work of the Citizens' Commission is organized around five workstreams: Finance, Human Resources for Health (HRH), Citizens' Engagement, Governance, and Technology. Five ToC workshops were conducted, one for each workstream. Individual workshop outputs were then brought together in two cross-workstream workshops where a sectoral Theory of Change for UHC was derived. Seventy-four participants, drawn from the Commission or invited for their expertise, and representing diverse stakeholders and sectors concerned with UHC, contributed to these workshops. A reimagined healthcare system achieves (1) enhanced transparency, accountability, and responsiveness; (2) improved quality of health services; (3) accessible, comprehensive, connected, and affordable care for all; (4) equitable, people-centered and safe health services; and (5) trust in the health system. For a mixed system like India's, achieving these high ideals will require all actors, public, private and civil society, to collaborate and bring about this transformation. During the consultation, paradigm shifts emerged, which were structural or systemic assumptions that were deemed necessary for the realization of all interventions. Critical points of consensus also emerged from the workshops, such as the need for citizen-centricity, greater efficiency in the use of public finances for health care, shifting to team-based managed care, empowerment of frontline health workers, the appropriate use of technology across all phases of patient care, and moving toward an articulation of positive health and wellbeing. Critical areas of contention that remained related to the role of the private sector, especially around financing and service delivery. Few issues for further consultation and research were noted, such as payment for performance across both public and private sectors, the use of accountability metrics across both public and private sectors, and the strategies for addressing structural barriers to realizing the proposed paradigm shifts. As the ToCs were developed in expert groups, citizens' consultations and consultations with administrative leaders were recommended to refine and ground the ToC, and therefore the roadmap to realize UHC, in people's lived reality.

## Introduction

The Lancet Citizens' Commission on Reimagining India's Health System is an ambitious, cross-sectoral endeavor to lay out the roadmap to achieving Universal Health Coverage (UHC) for the people of India. UHC is a critical path to achieving health equity in India and addressing the large unmet need for quality, affordable care, a need that was laid bare in the pandemic and which will worsen as the health impacts of climate change grow. The Commission's core vision is that such a structural change must be guided not only by the perspectives of policymakers and public health experts but equally through a consultative and participatory engagement with the diverse stakeholders of health care, key among whom are the citizens of the country. The Lancet Commission has brought together leaders from academia, the scientific community, civil society, and private healthcare to spearhead this effort. The Commission's work is structured across five workstreams: financing, governance, human resources, technology, and citizens' engagement. Each of these workstreams comprises a group of Commissioners and Fellows engaged in several ongoing research studies that address questions related to its scope. The Commission defines Universal Health Coverage as four defining principles: (1) UHC needs to cover all health concerns, (2) it is not limited to clinical treatment, but includes the prevention of mental and physical health problems and long-term care, (3) financial protections need to be present and available for all health-care costs, and finally (4) needs to support a health system that is accessible by all citizens for the same quality of care. Although the Commission recognizes the importance of social determinants of health, this is not within its current scope (1).

The Commission selected the Theory of Change methodology to prepare a roadmap to realize UHC in India. The Theory of Change (ToC) approach is rapidly gaining recognition in public health in relation to the design, implementation, and evaluation of health systems. The ToC approach identifies an outcome or goal and brings together stakeholders to identify causal pathways that would reach that goal. This mode of discussion opens up debates on the recommended actions, the path, and the underlying assumptions at play that make a causal pathway successful. TOC workshops provided forums for participants to not only interrogate pathways to achieving UHC within each workstream but also to derive synergies across workstreams. The ToC used a participatory approach by bringing together a range of stakeholders to agree on the impact, the final outcomes, and the intermediate outcomes that result from certain causal pathways. The purpose was not only to reach a consensus on the pathways but also to surface any apparent or underlying tensions. Subsequent consultations led to the development of a sectoral ToC map which included assumptions and interventions, as well as areas for further consultation and research. The assumptions are structural in nature and are referred to as paradigm shifts needed to achieve the desired outcomes and were intentionally and carefully crafted by participants in the workshop led by the Commissioners. All workstreams were not uniform in the depth of the thinking and consensus at the time of the ToC exercise, hence this paper is reflective of this status.

The Commission adopted a two-phase approach to developing the ToC map. In the first phase, each workstream developed its TOC maps, identifying the goals for that workstream, the interventions, associated outcomes, and pathways that lead to their intended goals. In the second phase, the ToC maps of all five workstreams were synthesized, and areas of convergence and discrepancy were identified. The entire process was participatory, with representation across different sectors.

## Materials and methods

### Participants

In total, 77 persons participated in one or more of the seven workshops. Each workshop included Commission members and other experts external to the Commission. Ultimately, 16 Commissioners, 17 Commission Fellows, and 41 external participants participated in this research (Appendix A). The participants were selected to ensure the representativeness of diverse sectors concerned with UHC (private, public, and civil society).

### Approach

**Figure 1:**
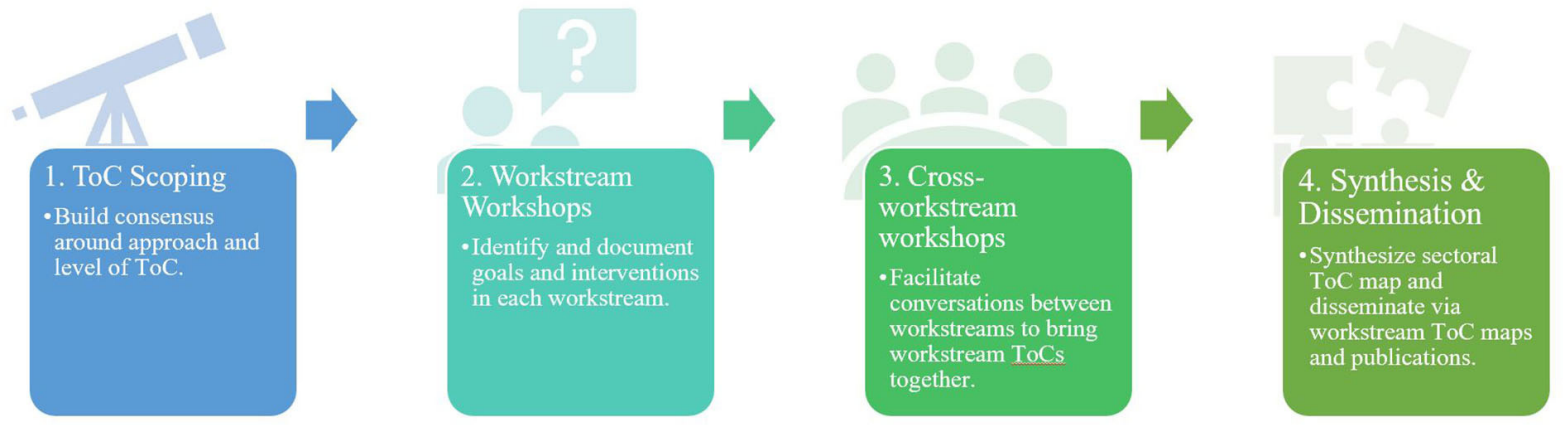
The theory of change process.

The ToC methodology followed a 5-step process (Figure 1): (1) ToC scope and consensus building; (2) workstream-specific workshop; (3) cross-workstream workshop; and a final (4) synthesis and dissemination. The process produced (1) workstream ToCs, (2) an overall ToC describing interventions and pathways to realize UHC; and (3) points of consensus and contention.

### Theory of change scoping

We reviewed the literature on the application of ToC models to achieve UHC in low- and middle-income countries and identified several articles either applying the ToC methodology to complex interventions (2–8) or articles that described efforts, other than the ToC methodology, to build frameworks or approaches to achieving UHC (9–11). Most ToC methodology papers focused on the creation, implementation, or integration of health programs (4, 5, 7, 8), especially those focusing on mental health programs (2, 3, 6). These papers provided approaches and tools that we could adapt to our methodology. Other articles described efforts to create UHC frameworks that could apply to an LMIC context. This included the UHC framework of Asian Development Bank that outlined indicators for measuring activities, impacts, and outcomes (10) or WHO's UHC framework that identified how health system building blocks and UHC domains could achieve the impacts required for UHC (9). None however adopted a ToC approach for UHC either in India or other LMICs. Since a ToC approach is primarily used for program mapping, determining the level at which we needed to set our goals, outcomes and interventions was critical. A granular approach pegged at a health facility's level, for instance, would be unable to accommodate varying contexts. At the same time, a macroscopic approach ran the danger of being too generic. Consultations with ten Commissioners led to the decision of calibrating the scope of the ToC to government policies and other structural issues that were identified as key obstacles to achieving UHC, and pertinent to all actors in the health sector (i.e., not limited to government stakeholders alone).

### Workstream theory of change workshops

All five workstream workshops were held virtually due to pandemic restrictions and followed the process of first identifying and articulating how each workstream would contribute to UHC through its impact statements, i.e., the overarching goals for that workstream TOC. These impact statements were used to identify intermediate outcomes, activities, and interventions. For instance, for the finance workstream, reducing and improving the value of out-of-pocket expenditures was identified as an intermediate outcome. A facilitator moderated the discussions by utilizing a Miro board, an online whiteboard with placeholders for all stages of the goal, interventions, intermediate outcomes, outcomes, and impact. These workshops were conducted between the period of August 2021 and October 2021. After each workshop, key findings were prepared and circulated to the participants for review and comments, and the workstream ToCs were revised and finalized (Appendix B).

### Cross-workstream workshops

The first cross-workstream workshop was held on January 10, 2022 virtually due to another COVID-19 wave in India. The virtual session limited the intended scope of the workshop, and we focused instead on presenting individual workstream TOCs and incorporating further revisions in preparation for the larger cross-workstream workshop to be held in person. A key outcome of these discussions was the articulation of specific paradigm shifts (Appendix C) that would need to undergird each workstream's ToC diagram. The proposed paradigm shifts refer to structural or conceptual changes that need to move the needle on the status quo in the architecture of the healthcare system.

The second cross-workstream workshop succeeded in bringing workstream participants, along with external experts, together in an in-person forum where workstream chairs were able to present their ToC, identify interlinkages with other work streams, and work to build a unified ToC. This workshop was conducted at the Indian Institute of Science in Bangalore on March 10, 2022. A total of 33 participants were drawn from the Commissioners (*n* = 15), Commission Fellows (*n* = 4), and other special invitees (*n* = 14). The workshop participants mapped out themes that cut across the workstreams, including paradigm shifts deemed common and essential from a UHC perspective. The facilitators identified areas of contention from these discussions, and Mentimeter, an anonymized polling software, was used to design a poll on 15 topics to identify areas of consensus and issues or questions where further research and consultation were required.

### Synthesis and dissemination

After the cross-workstream workshop, a summary report of the meeting was drafted and shared with the participants. The summary of the five workstream ToC documents and the unified ToC is presented in this paper as a final output of this research.

### Ethical approval

Ethical approval was obtained from the Catalyst Group Internal IEC Board on August 18, 2021. All workshops were conducted with the participant's consent, and all narrative data were de-identified.

## Results

The results describe the final synthesis of all workstream ToC workshops and a cross-sectoral ToC for realizing UHC in 10 years.

### Finance theory of change

The Finance Workstream Workshop included eleven participants. The focus of the finance workstream was to develop innovative ways to leverage the public sector to address market failures in the health system. The scope included exploring and addressing challenges related to the sources and utilization of health expenditures to maximize financial risk protection and to ensure an effective, equitable, reliable, and responsive health system for all. Three impacts were identified: (1) universal coverage of financial risk protection leading to (2) a reducing trend in out-of-pocket expenditures, and (3) preventing catastrophic health expenditures. The intermediate outcomes that followed were identified as (1) an increase in public funding allocation such that public systems would be strengthened and financial protections and appropriate incentives for private sector value-based care would also become available; (2) governance of the financing mechanisms; (3) improved management and utilization of allocated funds so that there is an improved capacity to spend current resources to improve public care services and provide financial protections; and (4) reductions in secondary and tertiary costs by (5) ensuring prompt and comprehensive treatment, especially for chronic illnesses which lead to catastrophic costs.

The finance workstream developed four overarching interventions that work together to ensure systemic capacities are in place to address the needs of the citizens, especially the most vulnerable. The first intervention is concerned with building political will to increase public funding. This is vital for the Indian healthcare system, whose current public spending is underwhelming. Public funding was considered essential by participants framing healthcare as a public good. Even when other participants framed healthcare as a market opportunity, there was consensus that public financing needed to redress market failures. The concern however, was that increased public funding may take time to materialize, and therefore the other interventions are necessary to continue to build systemic capacity despite the lack of public funding. The second intervention relates to building management incentives to improve financial efficiencies, which is key to utilizing current public funding to improve healthcare services and outcomes. The current administrative approach of providing block sums of money to states should incorporate effective management principles for allocation and spending. Innovative financing mechanisms such as pay for performance were cited as some private sector principles to be considered seriously to encourage better outcomes from public sector providers. The third relates to designing and implementing approaches to leverage insurance, allowing everyone to receive financial protection for health and prevent catastrophic health expenditures. As the health system hopefully transitions to a larger amount of public funding, it will continue to garner high out-of-pocket costs. Therefore, the fourth intervention focuses on improving the value generated from out-of-pocket expenditures, which would still allow for better health outcomes for the money being spent by consumers.

The underlying assumptions were that these pathways will lead to the desired results only if particular paradigm shifts occur (12). These include a shift from discrete markets to managed competition between multiple insurances and health providers. Managed competition would allow citizens to buy into insurance and receive care from providers bound to a payer looking to keep costs low and buyers healthy. Shifting the focus away from reducing out-of-pocket expenditures to the value derived from the expenditure and moving from an emphasis on better financial management solely within the public sector to ensuring cost-effectiveness and efficiency of financial resource use in the entire health system were the other essential paradigm shifts.

### Technology theory of change

There were twenty participants in the Technology Workstream ToC workshop. The scope of the workstream was primarily concerned with identifying ways of leveraging technology to empower the citizen in their journey from illness to health. The six impacts that the technology workstream would need to achieve are: (1) increased access to services; (2) improved quality of services; (3) improved diagnosis and prediction at the individual as well as population level; (4) improved prevention; (5) improved treatment and care, and (6) reduced technological inequities. The intermediate outcomes that the workstream identified include (1) continuous, comprehensive, and closed loop navigated care for citizens; (2) population-level continuum of care; (3) improved citizens' rights of access and information privacy; (4) reduced technological malpractice and harmful use; (5) increased adoption and use of technology in the health sector.

Five overarching interventions are needed to contribute to the goals of the technology workstream. The first intervention relates to reimagining health journeys through a citizen-centric approach. This approach would require developing technological aids for multiple touch points of care defined for various scenarios and settings. An integrated population-level delivery system for a continuum of care would need to be established. New-age, innovative data management systems that cater to all stakeholders in the system, from patient to provider, need to be set up. The second intervention relates to improving the productivity of existing health systems, commencing with digitizing citizens' health data in a flexible, secure and interoperable manner. Protocols, standards, and tools need to be in place to build an integrated digital health ecosystem that serves all other stakeholders in the sector. These technologies must then be promoted and disseminated to reach the wider public. We also need to ensure capacity building of personnel to utilize and leverage existing technology. Further, with data management being critical to this workstream, the third intervention relates to creating a holistic techno-legal framework: establishing regulatory and enforcement mechanisms and transparent, high-quality audit systems. There was consensus that a robust regulatory framework that could target malpractice and ensure transparency was required for such a comprehensive digital undertaking. Other citizen-centric objectives related to establishing flexibility and interoperability of technology platforms to ensure concealment of sensitive health records and user-friendliness. The fourth intervention involves promoting and governing technological experimentation as India has a large and diverse talent pool. A well-regulated but market-friendly platform will encourage experimentation and social businesses. Finally, the fifth intervention relates to innovations in medical technologies and devices such as vaccinations, pharmaceuticals, sensors, biomarkers, environmental surveillance and more.

The paradigm shifts required to build a reimagined digital infrastructure should progress from one of administrative convenience to citizen-centric journeys. Technology is needed to enable the health system to work for the citizens. This would mean connecting people to care, self-care, and community support even before they fall ill or have delayed diagnoses leading to medical crises and catastrophic health expenditure. It would also mean creating a system that reduces gaps and friction in the users' journey by offering digital mediums to transform siloed, disconnected interactions into a continuum of care. A significant focus of the health system has to be prevention and diagnosis, a paradigm shift from the current focus toward remedial actions. There was consensus that technology could play a critical role in improving preventive and diagnostic outcomes, especially through improved access, and make this challenge feasible, apart from continuing to play a role in improved treatment and care outcomes. Another necessary shift included digitally augmenting the capacities of the health workforce that would result in a paradigm shift of providing care away from hospitals and labs and in frontlines and homes. Given the human resource challenges in India, the workstream recommended the role of technology to mitigate errors and deficiencies and enhance human capabilities, including that of frontline health workers. Technology can offer digital solutions to augment HRH expertise and efficiency by limiting data collection for reporting purposes and focusing on data utilization by caregivers through intuitive digital data capture and usage. Technological innovation in the private sector should be fostered through open access, public platforms and protocols, and the public sector has a role to play here.

### Human resources for health theory of change

Eighteen participants representing a mix of internal and external experts were present for the Human Resources for Health workshop. Human resources for health (HRH) include all those persons whose primary work is to deliver health and health care (promotive, preventive, curative, rehabilitative and palliative). HRH in the Indian context spans both the allopathic medicine system and the AYUSH system and comprises a diverse range of actors, notably including a range of frontline health workers. The scope of this workstream is to propose a transparent system within the regulatory framework to nurture, enumerate, train, and equitably redistribute HRH at decentralized, hyper-local levels of governance, and to create a concerted push toward team-based healthcare through multi-pronged policies and a life-cycle approach for HRH.

To leverage this diverse array of HRH, the four impacts articulated include (1) the development of a competent and responsive workforce that functions through (2) interprofessional, comprehensive, and person-centric teams throughout the life cycle (3) sustainable production of qualified, competent, accountable, motivated and empathetic HRH for delivering healthcare services reaching every citizen. Finally, the ToC identifies (4) ownership and pride among local communities in human resources of health, which is key to achieving UHC. The intermediate outcomes are (1) positive and forward-looking healthcare teams that provide holistic care and focus on comprehensive primary, preventative care and palliative care; (2) policies that facilitate career progression, lifelong learning and performance development, growth and certification of competencies; and (3) engagement of local communities and the sector to understand the importance of team-based care, including active participation of individuals and family members in personal and community health.

The ToC for the HRH workstream has identified three overarching interventions to achieve the stated outcomes. Firstly, it is recommended that the overall approach to HRH be redefined, including the HRH requirement at the district level, to ensure equitable distribution, along with decentralizing planning, functions and responsibilities. This redefinition must happen politically, systemically, and culturally. It further requires establishing a transparent, whole-of-system regulatory framework to enumerate, train, distribute, and nurture HRH at decentralized, hyper-local levels of governance. Reforms are needed in pre-service education for HRH, including in topics such as public health, community engagement, and digital literacy. Front line workers (FLWs) need to be integrated into HRH structures, and institutional mechanisms for facilitating scientific, tech-enabled management of HRH need to be explored. A critical step toward UHC entails highlighting value-based care and creating a holistic environment where preventive and promotive healthcare is the first line of work with communities. The second set of interventions focuses on creating team-based managed healthcare provision through policies and incentives for specific roles. The success of such a team would depend on a multi-dimensional, evidence-based performance management framework that includes technical, managerial, social, psychological and cultural HRH competencies. This must include training for shifting social norms and building an effective interface with communities. Communities themselves must be viewed and mobilized as a talent pool for HRH. The third set of interventions follows from this: creating continuous HRH wellbeing with a focus on training, development and retention from a life-cycle approach where career progression, growth, pride, and ownership of work are enabled. Furthermore, institutional mechanisms for capturing comprehensive, anonymous feedback for HRH, including in health directorates at the state and district level and in the private sector, would ensure active listening and responsiveness for HRH.

The interventions listed are not radically different from recommendations in earlier discussions on reform in the country. However, the paradigm shifts are fundamental in achieving results. As COVID showed the country, the reliance on a resilient workforce requires an ecosystem for them to deliver their work competently with the support of the community they serve. The first shift identified relates to moving from highly specialized HRH to building strong team-based delivery at all levels of care with attention to cultural and language competencies. The focus must be on family medicine, and building pride and ownership within the family medicine/general medicine community. The second shift relates to the need for acknowledgment of all human resources within HRH policies and structures, including political acknowledgment, as opposed to the current emphasis on licensed practitioners. This acknowledgment includes mainstreaming the frontline health workforce and the large group of healthcare professionals under the allied health workforce. Doing so will alter the status quo to state-specific approaches to HRH production and deployment that is suitable to the local context at the district level as opposed to a centralized, top-down approach to allocation, placement, and distribution. The next shift relates to adopting a whole-of-system approach for HRH, measuring and fulfilling the need in both public and private sectors as opposed to sole focus on public sector training and pathways for HRH. The relationship between patients, communities and providers will shift from a hierarchical to a dynamic relationship through the empowerment of citizens who are considered partners in health who support and facilitate accountability of HRH, and engage with HRH in a mutually beneficial manner. These shifts refocus on a life-cycle approach for HRH that considers career progression essential to building growth opportunities, motivation, pride, and ownership of their work. It recognizes that capacities, competencies and performance management are continuous, lifelong themes that require grounding in the local context.

### Citizen's engagement theory of change

Fourteen participants, five commissioners, four commission fellows, and five external participants were present for the Citizens' Engagement ToC workshop. Citizens' engagement is a central focus of the Lancet Commission's vision that UHC must be designed with citizens at the center. The scope of this workstream involved defining an ecosystem where citizens identify their healthcare priorities and are engaged in planning, driving, implementing, and monitoring necessary actions in both the public and private health sectors. This demand-driven approach is essential to realizing a health system that is not just efficient but also democratic. Without proactive and purposeful citizens' engagement, health policies, systems and structures would remain deficient. Citizens' engagement is meant to establish a social contract based on genuine dialogue between those who control resources and those who lack resources and between those who provide access to health services and those that seek access to those services. There was consensus across the workstream that citizens' engagement must be based on a few essential values and principles, notably inclusion, equity, trust, dignity, compassion, agency, and rights.

Four areas of impact identified by the workstream included: (1) change in social norms, (2) empowered citizens who can make decisions about their health and influence policy, (3) ownership of the planning, design, implementation and monitoring of health services by the citizen public and, (4) shift to a “people-centric” attitude in the private and public sector. The intermediate outcomes include (1) equitable availability of information, (2) citizens' awareness about information and channels for advocacy, (3) strong public engagement mechanisms set up by governments, (4) community governance of health in both private and public sectors, (5) contextually relevant health services, (6) decentralization of power, authority and responsibility, (7) improved relations between providers and recipients, (8) grievance redressal mechanism in both public and private sectors.

Participants identified four overarching interventions to achieve the stated outcomes. They followed a trajectory of involving the community, starting from more passive activities to more active inclusion, including consulting/informing, collaborating, empowering, and finally enforcing accountability and community-led change. The first intervention described activities that consult and inform citizens. Some activities included estimating the needs and priorities of communities and using this as the basis to develop context-specific health education. Platforms and structures would need to be identified or established to amplify community voices with a special focus on improving the representation of marginalized groups and individuals. Some recommendations on the collaborative platforms that could be used to engage and organize citizens included local government, panchayats, co-operatives, unions, and self-help groups, with the belief that this would lead to stronger political will for UHC. Legal frameworks for consultation and information would also need to be strengthened. The second intervention relates to involving and collaborating with communities by bolstering systemic capacity for community engagement and community capacities for collaboration and participation. Processes to meaningfully monitor this intervention need to be set up and researched further. The third set of interventions focuses on empowering citizens for community-led actions for health. This means that communities need to be responsible for their health, develop solutions, take evidence-based decisions on health issues and challenges, and facilitate community engagement with health. It includes raising awareness among citizens on their health entitlements, available health services and the role of health providers. Finally, the fourth proposed intervention relates to creating accountability and trust in public and private health systems. Enhancing the scope of political engagement is a crucial step toward this, along with developing and disseminating performance reports on the functioning of various health systems. Communities should be able to conduct social audits of the health systems they use along principles of Community-Based Management (CBM). Establishing or strengthening the legal framework to protect and empower communities would also be essential, and a robust grievance redressal mechanism should penalize malpractice and negligence. However, care needs to be taken to ensure that caregivers are not unduly penalized.

The workstream identified a number of paradigm shifts to realize these impacts and outcomes. The shifts include moving from limited awareness and utilization of public services to an increased demand for quality health services, transforming citizens from being a passive audience to an actively engaged community, and shifting limited political will to promote citizens' engagement in health to developing a favorable policy and political environment for this. Furthermore, the onus of positive health outcomes should not solely be on the health system but should be shared between the citizens and the systems. Finally, there should be a shift toward full and free access and control of personal health records and system-performance data in multiple Indian languages in contrast to the prevailing pattern of limited or restricted access.

### Governance theory of change

A re-energized Indian healthcare system requires systemic, transformational change stemming from a renewed governance architecture. All fourteen workshop participants unanimously agreed that the governance framework should be characterized by a robust regulation architecture for all health care actors, smooth coordination across levels of government, civil society and the private sector, and increased accountability with the goal of high-quality healthcare for all citizens. There were debates on the scope of governance in the context of UHC to acknowledge and account for the diversity of actors in India and the factors that influence health outcomes well beyond the authority remits of the health ministry. In the end, the participants agreed on broadening the scope of governance from “the government” to governance across the health domain, including the private sector and civil society. The two impacts jointly articulated by participants include (1) high functioning public health institutions and (2) a participatory and accountable health system responsive to citizens' needs.

Key intermediate outcomes that were identified included (1) Clearly defined roles and responsibilities across all levels of government with the active participation of elected local governments in the delivery of healthcare; (2) Stronger and accountable regulatory institutions to create an enabling environment for the private sector while protecting citizen rights; (3) Improved data systems for health; (4) Lower absenteeism and more effective quality of care in government health facilities; and (5) Greater choice of care to patients (a more equitable and less predatorial health ecosystem for those in need of care).

While goals and intermediate outcomes were defined by the workstream, the interventions were not articulated within the workshop. The workstream instead proposed three pillars for research that would be conducted in the upcoming year to develop interventions for their ToC map. The first research pillar is related to strengthening the legal and regulatory architecture that empowers providers and protects patient rights. Participants noted that such an architecture would also be essential if technology was positioned to be an enabler that can transform accessibility and affordability. Data security and data sharing policies would need to be emphasized when researching the governance regulatory architecture. The second research pillar relates to federalism, and the way in which center-state dynamics shape the delivery and accountability of the public health system. In India's constitutionally mandated federal system, health is a State (sub-national) subject. Some aspects of public health policy and programming are shared with the center, while some functions for health delivery are devolved to local governments. From a first principles perspective, this federal structure is necessary to accommodate the variety and diversity of socio-economic pathways across India's states. The governance challenge is delineating roles and responsibilities, i.e., funds, functions, and functionaries across levels of government in a manner such that roles and responsibilities are aligned to the constitutionally mandated responsibilities of center-state (federal-local) and that there are clear lines of accountability. The third research pillar related to accountability: how do institutional dynamics, norms, perceptions and relationships of trust shape accountability.

Participants noted that there are government requirements for strengthening community accountability mechanisms, including for primary, secondary and tertiary healthcare in both the public and private sector. The group also discussed interventions in the governance of financing mechanisms, affirming that it would need to promote a healthy mix of public-private roles, which is context specific. While current governance interventions are skewed toward the public sector, the private sector is left wanting. In mixed health systems, where the high proportions of in-patient care happen in small private hospitals, particularly among the poor people, governance architecture will need significant strengthening. There are some efforts in this direction through *Rogi Kalyan Samitis* (13); however, implementing these governance mechanisms would need a high degree of resources and planning. The governance workstream determined these goals and areas of intervention through workshops but it is still undertaking further research to clearly articulate concrete interventions.

A paradigm shift for governance is the emphasis on the Right to Health, a fundamental right guaranteed to every citizen of India under Article 21 of the Constitution of India. Another paradigm shift would include moving from a centrally designed and funded governance system to decentralized, responsive governance that allows for planning, prioritization, and implementation by state and local authorities. This would include a strategic, evidence-based framework connected to a set of value-based outcomes instead of focusing on managerial, input-based metrics. This recognizes that governance is about remaking organizational culture as a whole without merely focusing on input-oriented rules. Other shifts include moving to a results-based system with contextualized designing and delivery of primary healthcare as a comprehensive service. This moves away from solely managing the public sector infrastructure through standardized protocols.

### Theory of change for universal health coverage

**Figure 2:**
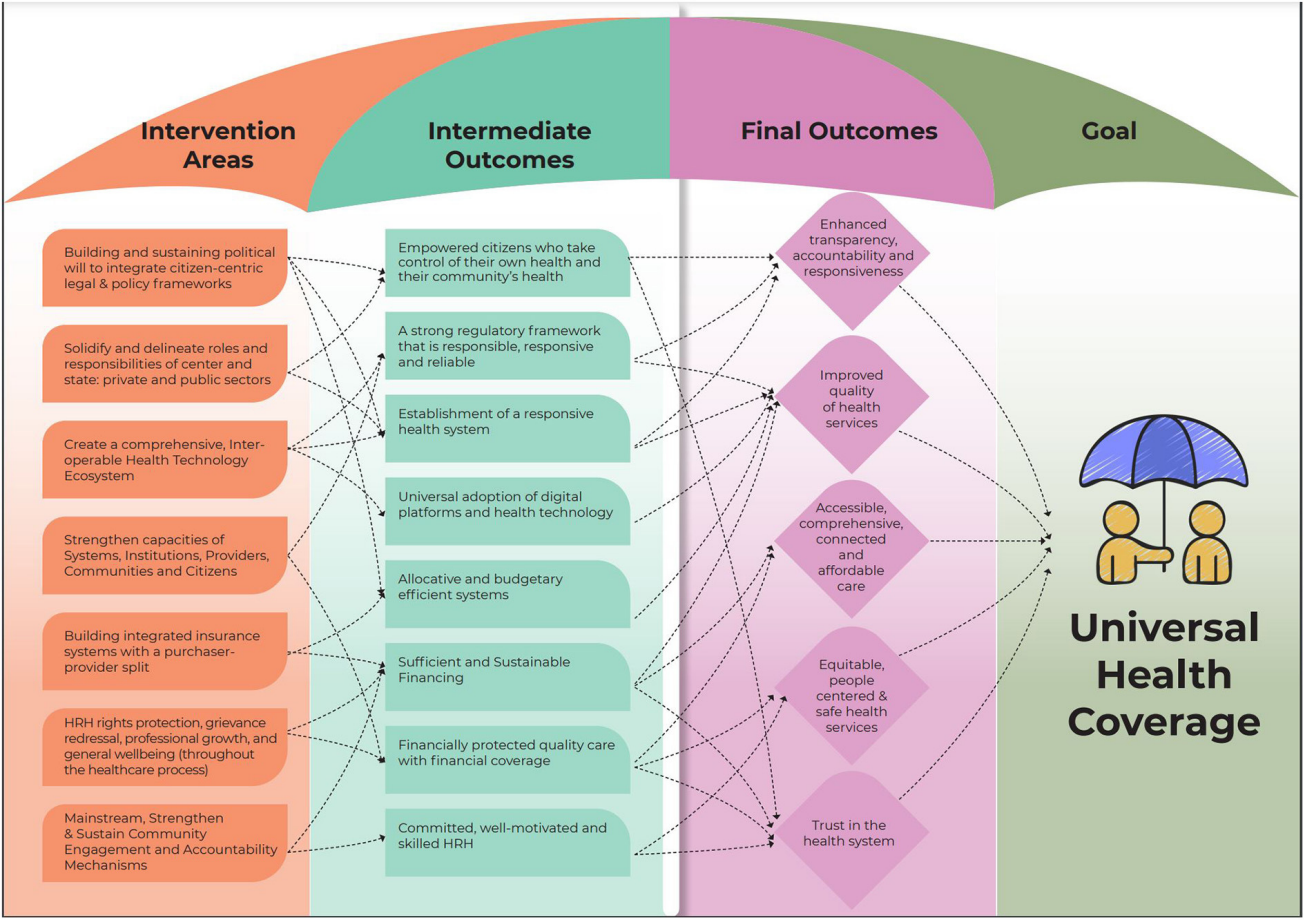
Cross-sectoral ToC Map to achieve UHC.

Clear trends emerged from the workstream ToC consultations that informed the ToC roadmap for UHC (Figure 2). This ToC roadmap for UHC synthesizes the information gathered throughout the workshops and frames the pathways toward the larger goal of UHC. The goal of UHC was adopted from WHO's articulation (13), which emphasizes quality health care for all citizens without financial hardship and covers the full spectrum of essential health services, from health promotion to prevention, treatment, rehabilitation, and palliative care across the life course, and was unanimously endorsed. Additionally, the possibility of UHC being framed through a rights-based lens was also considered. Further, the Commission also acknowledged the importance of the Astana Declaration in 2018, which emphasizes the state's responsibility to “make available primary health care that enables every person, everywhere to exercise their fundamental right to health” (14).

The final ToC proposed that UHC would lead to the following impacts: (1) enhanced transparency, accountability and responsiveness; (2) assured quality of health services; (3) accessible, comprehensive, connected and affordable care for all; (4) equitable, people-centered and safe health services; and (5) trust in the health system. For a mixed and fragmented system like India's, achieving these high ideals will require all actors, public, private and civil society, to collaborate to bring about transformation. The management of the pandemic provided an opportunity to innovate with technology being used extensively, indicating its potential for such a transformation.

To achieve UHC, participants identified a set of intermediate outcomes: (1) a robust regulatory framework that is responsible and reliable, (2) sufficient and sustainable financing and, (3) efficient systems for budgeting and allocation, (4) universal adoption of digital platforms and health technology, (5) committed, well-motivated and skilled HRH, (6) empowered citizens who take control of their health and their community's health, (7) financially protected quality care with financial coverage, and (8) establishment of a responsive health system. The workstream interventions have been synthesized into interventions which intersect and create synergies to reach their shared goals of UHC. The cross-sectoral interventions include (1) building and sustaining political will to integrate citizen-centric legal and policy frameworks; (2) solidifying and delineating roles and responsibilities of center and state; private and public sectors; (3) creating a comprehensive, interoperable health technology ecosystem; (4) strengthening capacities of systems, institutions, providers, communities and citizens; (5) building insurance systems with a purchaser-provider split, where non-competing tax-financed public insurance agencies can purchase care from a mix of public and private providers, thereby creating healthy competition between providers to offer better quality services and better health outcomes; (6) HRH rights protection, grievance redressal, professional growth, and general wellbeing (throughout the healthcare process); and (7) mainstreaming, strengthening and sustaining community engagement and accountability mechanisms. Each of these interventions contributes to multiple intermediate outcomes.

Several paradigm shifts were identified to realize UHC (Table 1). First, the healthcare system must shift its goal from being reactive and focusing on illness and disease to comprehensive wellbeing journeys for everyone. This would mean that the health ecosystem is responsive to a citizen's journey from preventing illness to receiving treatment and recovering health when sick, and shifts from siloed transactions to a healthcare continuum. Second, the health system must shift from generalized system-level surveillance to decentralized population and people-centered health surveillance to lead to more appropriate health care for each population. Third, there is a need to recast the role of citizens in the healthcare system, from being passive beneficiaries to active partners in a democratically representative system. There is a need to explore through community studies the potential levers and appropriate, acceptable platforms for citizens to engage in the health systems meaningfully. Fourth, there needs to be a paradigm shift in the generation of HRH from its current traditional emphasis on qualifications and hierarchies to skill-based and team-based approach to healthcare. Fifth, the estimation of HRH needs for each population must shift from focusing on the public sector to a whole-of-system approach, measuring and fulfilling the need in both public and private sectors. The plurality of the health workforce presents a unique opportunity to address HRH gaps and competencies, for example, through the formalization of ASHAs into the health system as justly compensated professionals and pathways for informal and rural medical practitioners to upskill themselves. Nurse practitioners and AYUSH practitioners should be empowered to both run tests and prescribe common medications in Primary Health Centers (PHCs). In order to ensure people-centered healthcare, the sixth paradigm shift requires governance to move from higher-level, centralized ministries to decentralized, community-led institutions. Centralized ministries predominantly manage only the public sector resulting in the recruitment of HRH for PHCs, and focus on input-based metrics for monitoring and evaluating public facilities (such as the number of PHCs).

**Table 1:** Paradigm shifts required to achieve universal health care.

**From**	**To**
Reactive healthcare focusing solely on illness and disease, and siloed transactions.	Comprehensive wellbeing journeys for citizens, emphasizing a continuum of care right from preventing illness to receiving treatment and recovering and maintaining good health.
Generalized system-level surveillance	Decentralized population and community level surveillance and proactive strategies.
Citizens being passive actors in the healthcare system	An active engagement of citizens in a democratically representative health system.
Sole focus on HRH training and employment in the public sector	A whole-of-system approach for HRH, measuring and fulfilling needs in both public and private sectors.
Higher-level, centralized ministries predominantly managing the public sector through input-based metrics (such as the number of PHCs)	Decentralized governance and decision-making in community-led institutions; remaking organizational culture and behavior.

In the final part of the workshop, a number of key issues which had arisen during its course were formulated into an anonymous opinion poll (Table 2). There was agreement on most of the 14 statements exhibited in the poll but also disagreement on three key points. There was consensus that while the primary responsibility of healthcare lies with the government, particularly stewardship, the accountability and financing of UHC was a diverse and joint endeavor involving the public and private sector and that citizens should be free to choose their service provider regardless of payment terms. Primary care must be significantly strengthened to be a gateway to accessing specialized care, and bypassing primary care would remove the protection against financial risk provided by the state. This sentiment is aligned with the concept of managed care organizations. There was consensus that healthcare quality needed as much attention as expanding access. The role of technology was uncontested in terms of improving access and efficiency, and there was consensus on the paramount importance of privacy of personal health data and the need to address inequities in digital literacy and access. However, there was contention on the way in which this digital infrastructure would be set up and its governance framework, and there was consensus that self-regulation was not an option and that an independent regulator was required. Only half of the participants felt that performance-based payments should be the predominant way to pay for healthcare, or that public and private providers should compete for public funds for health care delivery.

**Table 2:** Anonymous poll results on contended issues from the 2nd cross-workstream workshop.

	**Agree**	**Disagree**	**Can't Say**
1. The primary responsibility of healthcare lies with the government	26	7	1
2. Citizens should be free to choose the service provider (public, private, civil society) regardless of whether they are paying for service	28	6	0
3. While eliminating OOPE is ideal, in the short to medium term, getting better value for OOPE is more important	30	2	2
4. Digitally empowered healthcare workers can deliver better quality of care	21	4	9
5. Anonymized digital health records should be openly accessible for monitoring and evaluation of services	26	3	4
6. Self-regulation of technology is the most effective strategy for regulation as opposed to external regulation	3	26	5
7. Allopathic doctors are not necessary for primary healthcare (ex. Nurse practitioners/AYUSH should be empowered to run PHCs and prescribe)	22	9	3
8. Performance-based payments should be the predominant way to pay for healthcare	14	15	5
9. HR should be hired, managed, and regulated by local/district level authorities, NOT by state or central government	25	5	4
10. ASHAs should be formalized into the health system and salaried	26	7	1
11. Primary care must be the gateway to access specialized care (i.e., bypassing primary care removes financial risk protection by the state)	30	1	3
12. Informal/Rural medical practitioners should be offered a pathway to upskill and become members of the formal health workforce	27	6	1
13. We need an independent regulator for health that is separate from the health department (e.g. TRAI, SEBI)	33	0	1
14. Public and private providers should compete for public funds for healthcare delivery	18	15	1

## Discussion

This paper describes a comprehensive, multi-step process to develop a Theory of Change for Universal Health Coverage in India, sponsored by the Lancet Citizens Commission on re-imagining India's health care system. The methodology involved conducting seven workshops with a total of 77 unique participants, supplemented with more than fifty individual or small group consultations conducted between the period of April 2021 to April 2022.

The ToC approach is useful to delineate and clarify causal pathways as well as highlight underlying assumptions that feed into achieving UHC in incremental, interconnected steps. It is a whole-of-system approach that begins with the end goal and works backwards to determine necessary actions. We describe the scope, impact, intermediary outcomes, interventions and paradigm shifts required for the realization of UHC across each of the five work-streams of the Commission, viz., governance, citizens engagement, financing, HRH and technology. The resulting cross-sectoral syntheses identified a number of overarching paradigm shifts that provide a framework for the specific interventions needed within each Commission's workstream. However, we acknowledge limitations and challenges. Firstly, the Lancet's Citizen Commission's scope is limited to the traditional health sector alone. The Commission recognizes the critical influence of social, political, and economic conditions outside the sphere of clinical care that can affect the burden of health on the population, but has *a priori* focused on UHC. Secondly, while the Commission was structured around workstreams which was helpful for efficiency and focus, this led to more supply-side deliberations rather than a citizen-centric approach to health, creating potential biases in interventions. Thirdly, the unfortunate timing of this research with the COVID pandemic resulted in six out of seven workshops being conducted virtually, limiting the time and depth of discussions. Fourth, some key discussion areas were missing, for example, those related to health products like drugs, diagnostics, and the supply chain. A fifth limitation was the lack of representation in the workshops of representatives from the private sector and Indian systems of medicine. Finally, this ToC assumes that citizen behavior would organically adapt to the proposed interventions but this may not materialize. For example, introducing a managed care approach with mandatory first consultation in primary care rather than allowing citizens to reach out to specialists themselves may not be culturally acceptable.

Discussions on how to move toward UHC have been ongoing for decades, with limited progress despite several robust attempts. The consultations around the ToC described in this paper have represented some of these ongoing debates which, when unresolved, create barriers that stop the system from evolving toward a common good. In some instances, even though most stakeholders agree on a specific paradigm shift or intervention, for example, that there should be a formalization of ASHAs, there still seems to be policy inaction. In other instances, there are significant differences in opinion, at least amongst expert stakeholders, on certain issues, in particular those concerning the modalities for financing and regulating the private sector, the deployment of market-based approaches, the role of commercial insurance and the feasibility and acceptability of performance-based payments. Many participants were apprehensive about framing health interventions as market interventions and raised the issue that healthcare should be viewed as a public good rather than a market opportunity. This issue remained contentious and left for further debate. It would require sensitive articulation to ensure acceptance and implementation. Concerns about the scale of corruption in healthcare continue to remain poorly addressed either by scholarship or debate and discourses.

The documented process is the first milestone for the Lancet Commission to build a comprehensive, coherent roadmap for UHC. The findings identify the key questions and issues that need further exploration to reinforce the causal pathways or alter them to address contextual factors. Many of these limitations and assumptions that shaped the paradigm shifts need to be further tested, both through citizen consultations and pilot evaluations. As a consequence of the ToC workshops, the Commission is engaged in a series of primary research studies including a large, nationally representative population survey to elicit citizen views and preferences; a qualitative study of key informants concerned with UHC policy on the strategies to address the structural barriers toward implementing the paradigm shifts; and case studies of districts purposively selected to reflect a range of performance indicators on a newly developed UHC index. The findings of these diverse studies will be triangulated in due course to test the assumptions and revise the ToC presented in this paper.

## Data availability statement

The original contributions presented in the study are included in the article/Supplementary material, further inquiries can be directed to the corresponding author/s.

## Ethics statement

The studies involving human participants were reviewed and approved by Catalyst Foundation Institutional Ethics Committee. The patients/participants provided their written informed consent to participate in this study.

## Author contributions

VP, SK, and AC conceptualized and designed the study. NB, AC, and RG drafted the initial manuscript and reviewed and revised the manuscript. AC and NB led the analysis. PJ, NM, TN, MC, and VP participated in the study design and critically reviewed the manuscript. All authors approved the final manuscript as submitted and agreed to be accountable for all aspects of the work.

## Funding

Funding for this study and publication was provided by Harvard Global Research Support Centre India and implemented by Catalyst Management Services.

## Conflict of interest

The authors declare that the research was conducted in the absence of any commercial or financial relationships that could be construed as a potential conflict of interest.

## Publisher's note

All claims expressed in this article are solely those of the authors and do not necessarily represent those of their affiliated organizations, or those of the publisher, the editors and the reviewers. Any product that may be evaluated in this article, or claim that may be made by its manufacturer, is not guaranteed or endorsed by the publisher.
