# Supplementary material for: A theory of change roadmap for universal health coverage in India
Source: Front Public Health. 2022 Dec 1;10:1040913. doi: 10.3389/fpubh.2022.1040913 (PMC9751860; doi:10.3389/fpubh.2022.1040913)
Supplement: Supplementary file 1 [file Data_Sheet_1.docx]

Supplementary Materials

# Appendix A. Participants of the Lancet Theory of Change Process

| Commissioners (n=18) | | |
| --- | --- | --- |
| Tarun Khanna (Harvard Medical School) | Nachiket Mor (The Banyan Academy of Leadership in Mental Health) | Thelma Narayan (SOCHARA) |
| Dr. Leila Varkey (Independent Public Health Researcher) | Poonam Mutterja (Population Foundation of India) | Sapna Desai (Population Council) |
| Mirai Chatterjee (Self-Employed Women’s Association (SEWA)) | Sujata Rao (Former Secretary of Health and Family Welfare, Government of India) | Vikram Patel (Harvard Medical School) |
| Yamini Aiyar (Centre for Policy Research) | Vijay Chandru Indian (Institute of Science) | Sharad Sharma (ISPIRT Foundation) |
| Arnab Mukherji (Centre for Public Policy, IIM Bangalore) | Gagandeep Kang (Christian Medical College) | Kiran Mazumdar Shaw (Biocon) |
| Preethi John (Chitkara Global Health Institute) | Sandhya Venkateswaran (Centre for Social and Economic Progress) | Atul Gupta (University of Pennsylvania) |
| **Fellows (n= 16)** | | |
| J Pratheeba (Health Systems Transformation Platform (HSTP)) | Tejasvi Ravi (Healthcare investor @ Lightrock India) | Mekhala Krishnamurthy (Centre for Policy Research) |
| Bindu Ananth (Dvara Research) | Neela Saldanha (Ashoka University) | Tejasvi Ravi (Lightrock India) |
| Hasna Ashraf (Dvara Research) | Aarti Nagarkar | Alok Vajpeyi (Population Foundation of India) |
| Sanghamitra Singh (Population Foundation of India) | Anjali Nambiar (Dvara Research) | Dipanwita Sengupta (Christian Medical College) |
| Radhika Gore (Director of Research at the Family Health Centers at NYU Langone (FHC) | Raghu Dharmaraju (ARTPARK (AI & Robotics Technology Park) at IISc) | Abijith Biji (PhD student at Icahn School of Medicine at Mount Sinai) |
| Sudheer Kumar Shukla (Lancet Fellow) |  |  |
| **External Participants (n= 43)** | | |
| Anant Bhan (Sangath) | Armida Fernandez (Institute for Global Health) | Ashish Satav (MAHAN Trust) |
| Bijit Roy (PFI) | Yogesh Kalkonde (Sangwari, Surguja, Chhattisgarh) | Ved Arya (SRIJAN, Buddha Fellowship Program) |
| Dr. Shrey Desai (SEWA) | Aditya Unnikrishnan (Centre for Policy Research) | Ragini R Munjuluri (Internet Freedom Foundation) |
| Rajeev Sandanandan (Health Systems Transformation Platform - HSTP) | Sonakshi Sharma (Centre for Policy Research) | Sonalini Khatrapal (Asian Development Bank) |
| Sundararaman T (former Executive Director of National Health Systems Resource Centre) | Sunil Nandraj (Independent Expert) | Anup Karan (Public Health Foundation of India) |
| Chandrakant Lahariya (WHO) | Darshan Shankar (Foundation for Revitalization of Local Health Traditions) | Dr. S Saravana Kumar (Dr Mehta's Hospitals) |
| Mahesh Mathpati (London School of Hygiene and Tropical Medicine) | Karthikeyan K (Basic Health Services) | Dr. C.M. Bhagat (Bhagat Hospital) |
| Sitanshi Sharma (Society for Applied Studies) | Pavitra Mohan (India Development Review) | Rajani Ved (Bill and Melinda Gates Foundation) |
| Sanjay Nagral (Surgeon) | Sanjay Zodpey (Public Health Foundation of India) | JVR Prasad Rao (Former UN SG Special Envoy on AIDS in Asia Pacific and former Health Secretary, GoI) |
| Satchit Balsari (Harvard) | Vidhya R (Bangalore Baptist Hospital) | Sharad Iyengar (Action Research & Training for Health (ARTH)) |
| NS Prashanth ([Institute of Public Health Bangalore](http://www.iphindia.org/)) | Atul Kotwal (National Health Systems Resource Centre (NHSRC)) | Ajay Nair ([Swasth Digital Health Foundation](http://www.swasth.app/)) |
| Nerges Mistry (The Foundation for Medical Research) | Ajay Bakshi (NeuranceAI) | Darez Ahamed (Mission Director,  National Health Mission TN) |
| Sunita Nadhamuni (Digital Lifecare  Dell Technologies) | Sehj Kashyap (Harvard) | Srikant Nadhamuni (Independent Consultant) |
| Lilianna Bagnoli (Dimagi) | Sriram A S (Consultant) | Sethuraman (Argusoft) |
| Shayoni Mazumdar (Dimagi) |  |  |

# Appendix B. Workstream Theory of Change Diagrams (Figures 2 - 7)

Figure 2. Finance workstream ToC diagram


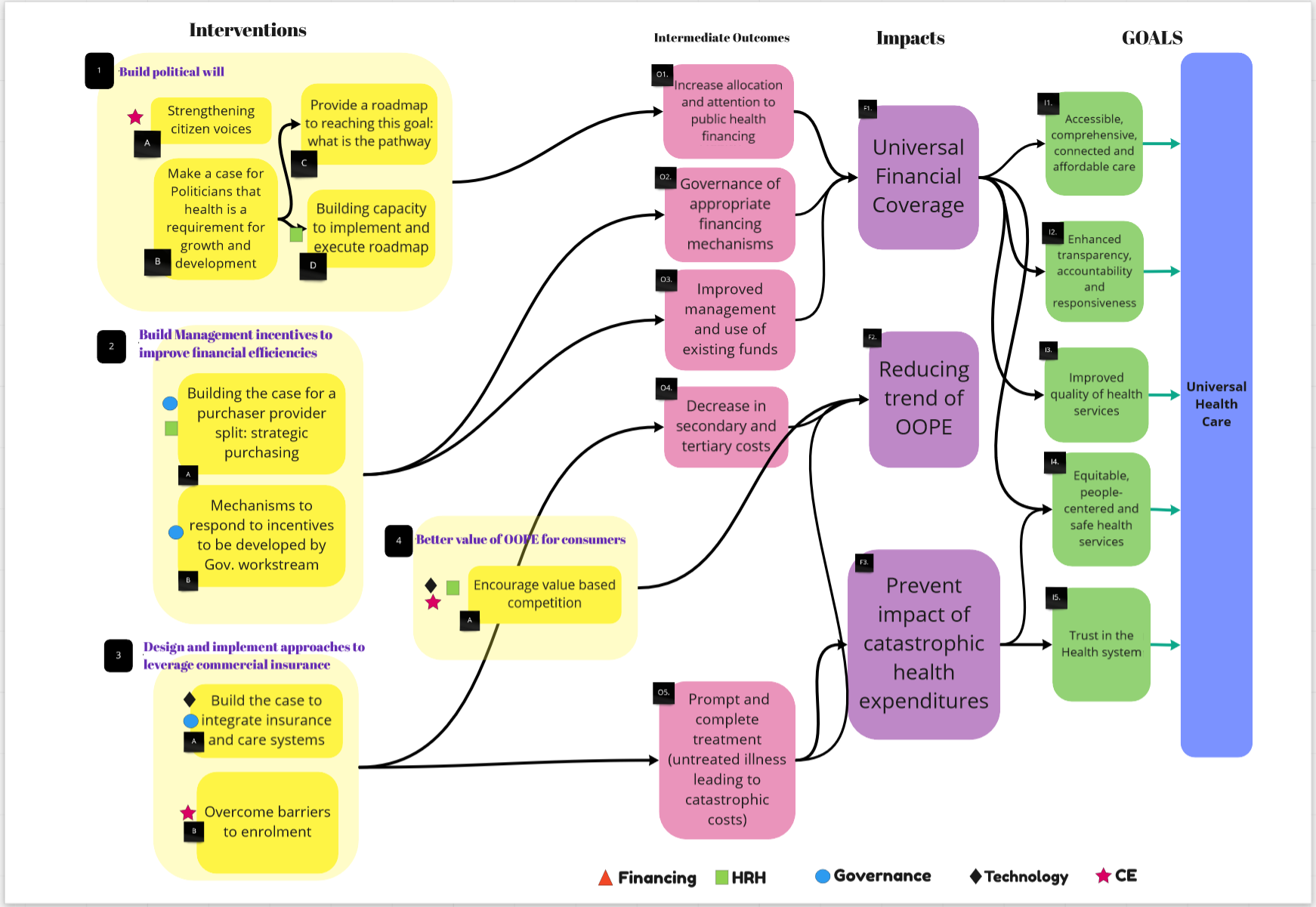


Figure 3. Technology workstream ToC diagram


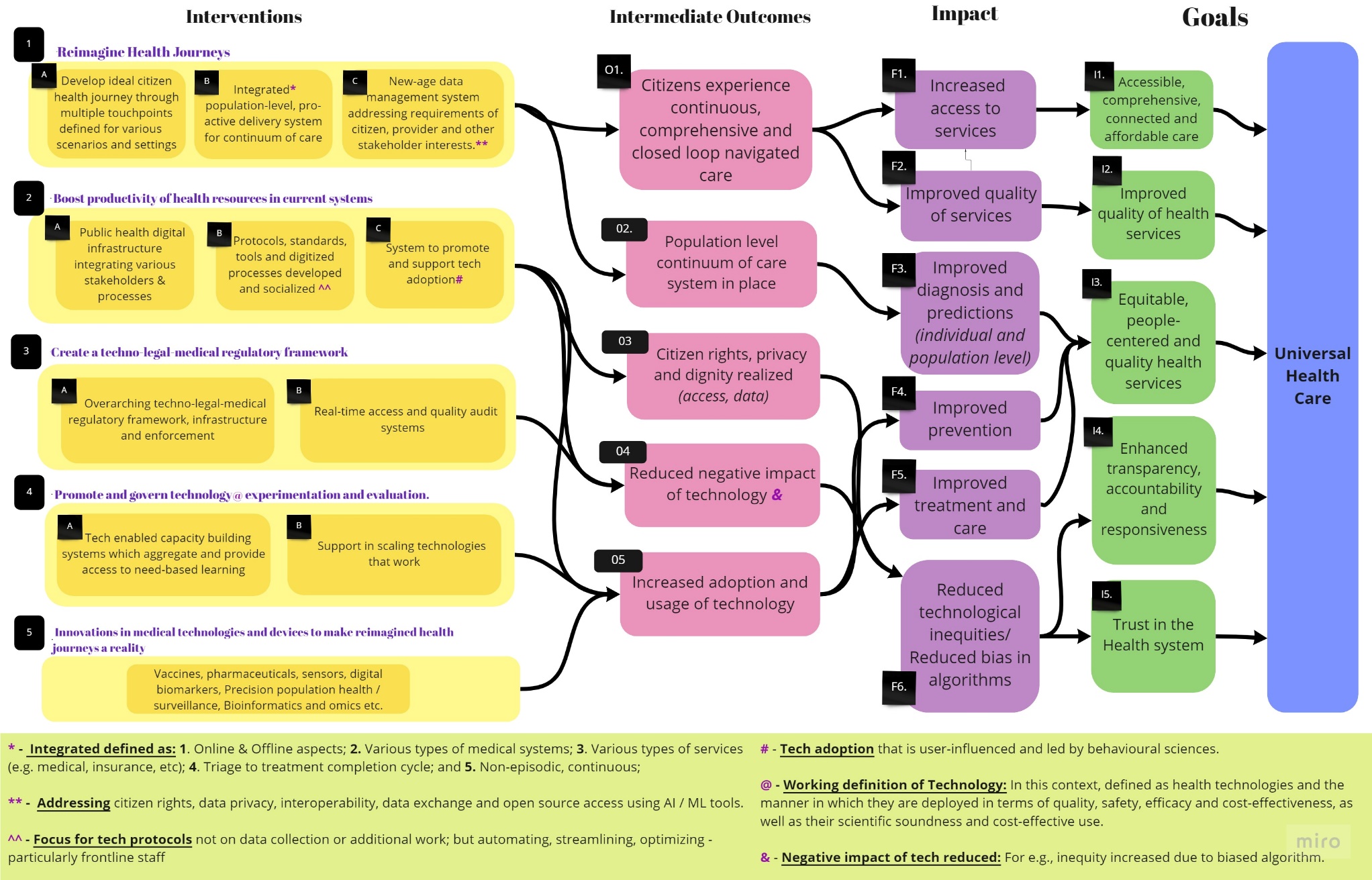


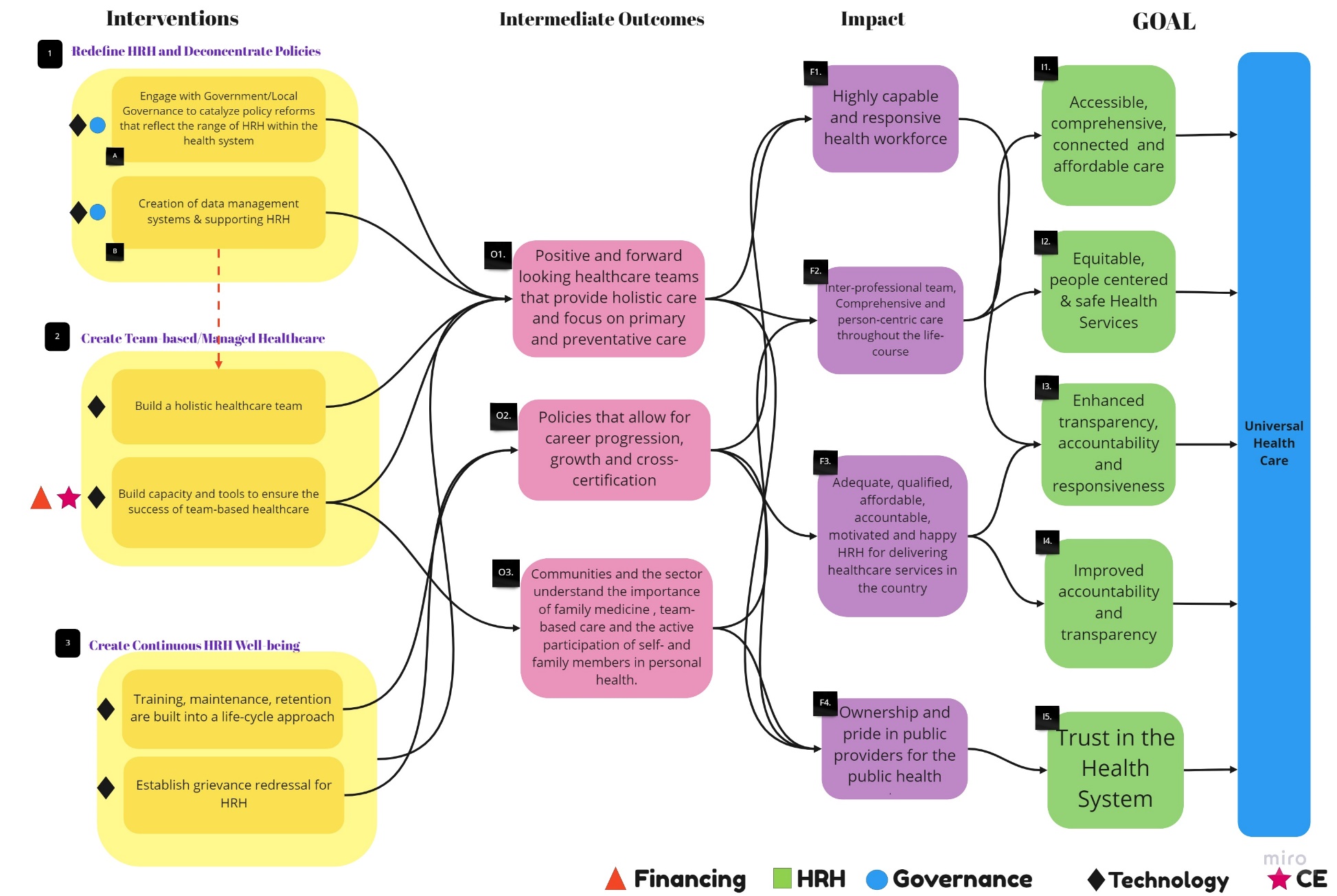
Figure 4. HRH ToC diagram


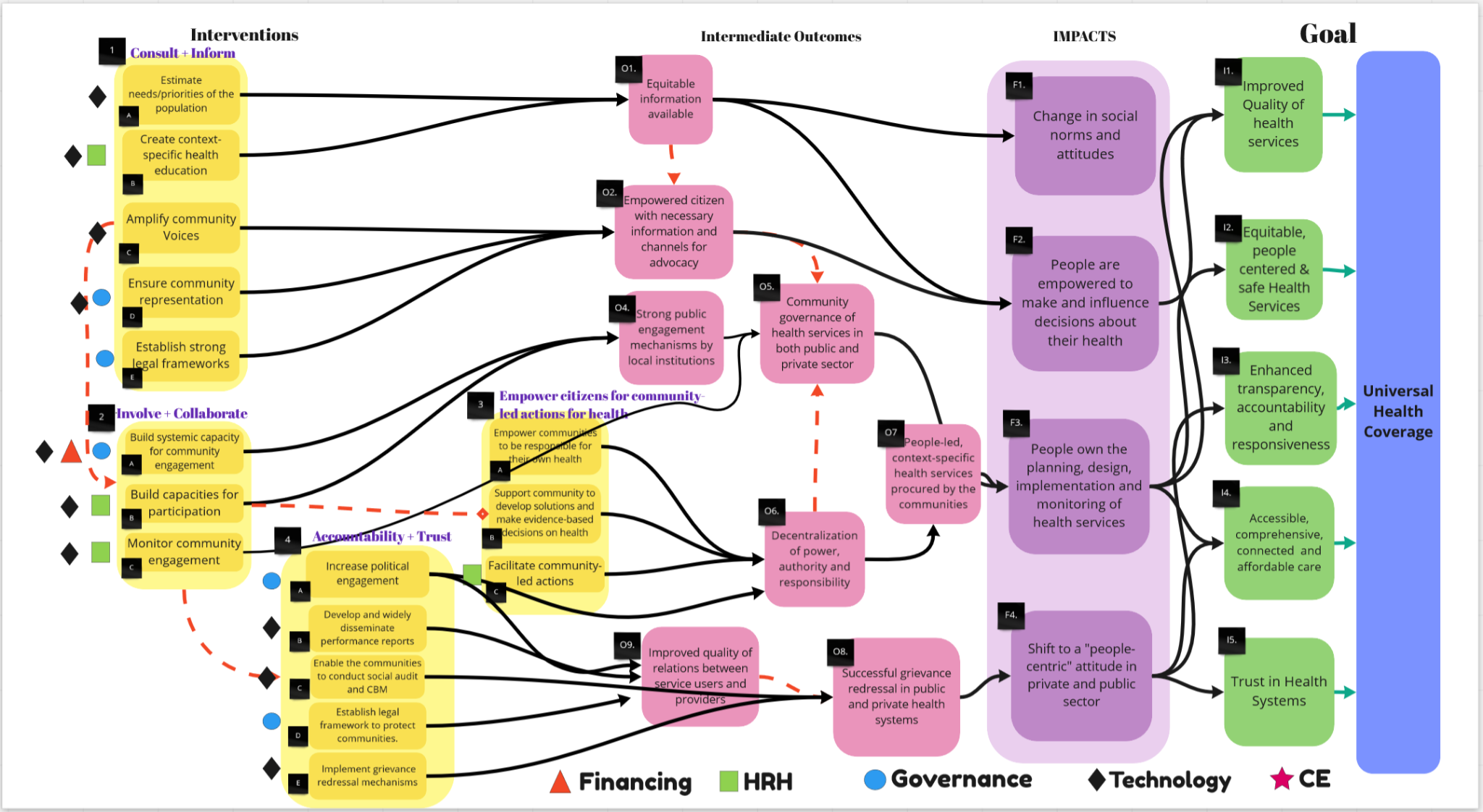
Figure 5. Citizens’ Engagement ToC diagram

Figure 6. Governance workstream ToC diagram


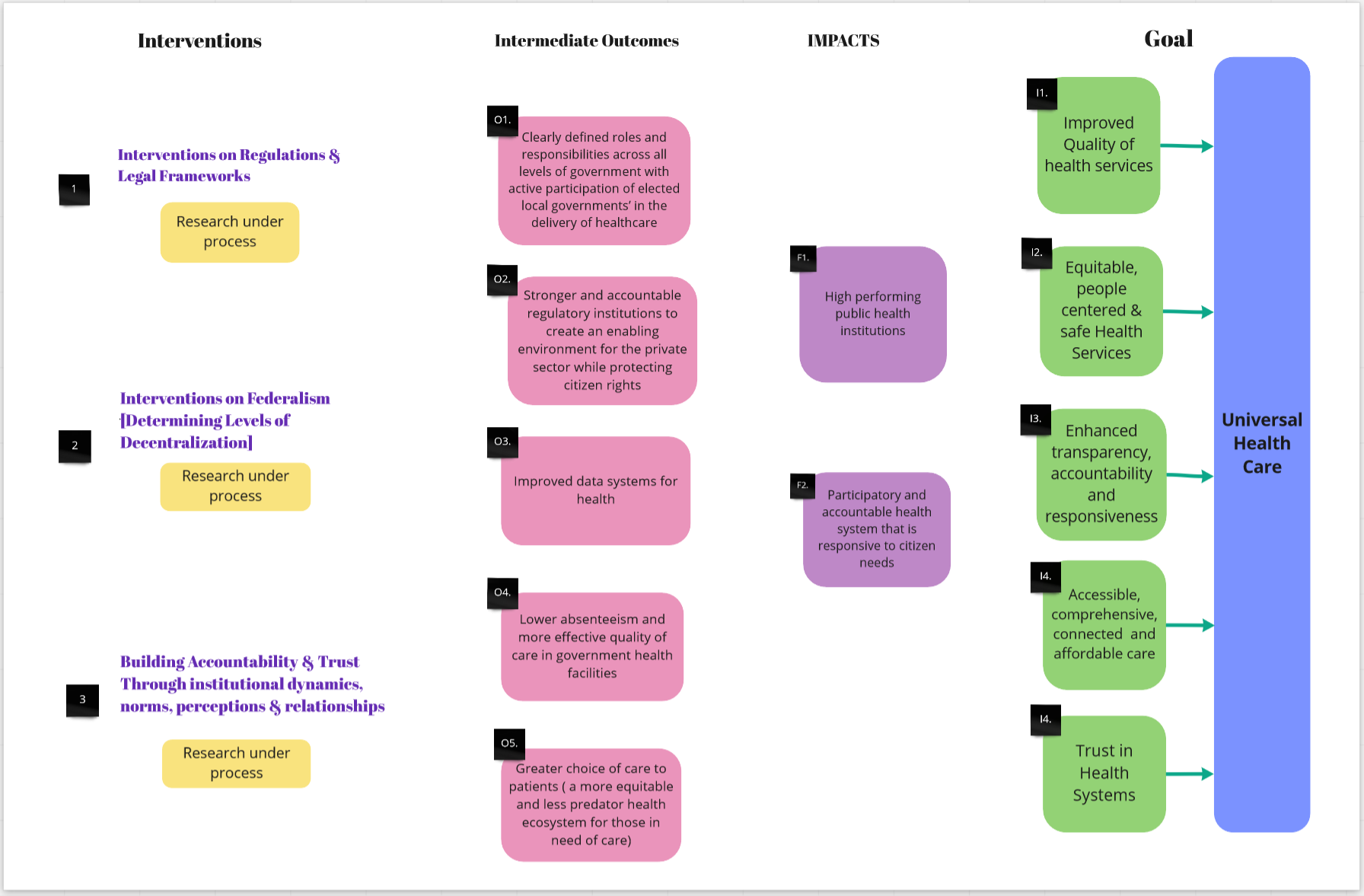


# Appendix C. Workstream Paradigm Shifts

| **Financing** | | | | **Citizens' Engagement** | | | |
| --- | --- | --- | --- | --- | --- | --- | --- |
| **From** | |  | **To** | **From** | |  | **To** |
| 1 | Offering multiple small and fragmented tax-financed purchasing schemes for primary and secondary care to population subsets. | 1.1 | Redirecting these investments towards strengthening the ten essential public health services (CDC, 2020). | 1 | Limited awareness and uptake of services in public health system | 1 | Increased demand for quality healthcare and utilization of public services |
|  |  | 1.2 | Redirecting these investments towards strengthening secondary care infrastructure in underserved areas. | 2 | Citizens are passive actors in the healthcare system | 2 | Active engagement of citizens in the health system |
|  |  | 1.3 | Replacing them with a single tax-financed scheme that offers financial protection against very rare and very high-cost conditions to the entire population. | 3 | Limited political will to promote citizens ’engagement in health. | 3 | Favorable policy and political environment, with a commitment to promoting citizen’s engagement in health |
| 2 | Completely ignoring out of pocket expenditures | 2.1 | Ensuring universal access to, even if not universal utilisation of, well-designed and reasonably priced financial instruments for financial protection through tools such as health savings accounts, commercial insurance, and “public options”. | 4 | Inequitable access to and the response of healthcare services for vulnerable groups | 4 | Equity, focused and responsive healthcare resources and delivery |
|  |  | 2.2 | Improving the value derived by citizens from OOP on primary and secondary care. | 5 | Positive health outcomes are seen as the sole responsibility of the health system | 5 | Both citizens and health systems are equally responsible for positive health outcomes |
| 3 | Commercial insurers and private healthcare providers competing in distinct markets. | 3 | Enabling competition between multiple integrated providers. | 6 | Limited or no access of citizens to personal health records and system performance data | 6 | Full and free access and control of personal health records and performance data, available in a language that can be comprehended easily by citizens. |
| **Technology** | | | |  | **Governance** | | |
| 1 | Citizen journeys shaped by administrative convenience. | 1 | Designing a health system for citizen-centered health journeys. | 1 | From government policy and ministries predominantly managing the public sector through input-based metrics. | 1 | Shifting governance regimes toward a decentralized, community-centered outcomes approach. Specifically recognizing that governance is about remaking organizational culture, not through tighter input-oriented rules. |
| 2 | Focusing on treat of illness as healthcare. | 2 | A system that promotes health & wellness, prevention. | 2 | National-Centrally designed and funded (schemes and plans) governance | 2 | Decentralized, responsive governance. Allows for planning, prioritization, and implementation by state and local authorities. This would include a strategic, evidence-based framework connected to a set of (value-based) outcomes. |
| 3 | Siloed, unconnected interactions within healthcare sectors. | 3 | A continuum of care and follow-ups within all healthcare sectors. | 3 | Government focused on managing only the public sector using nationally standardised approaches. | 3 | Governing the entire health system with a focus on outcomes and flexible designs which are responsive to the local context. |
| 4 | Services focused in hospitals and labs. | 4 | Services and touchpoints prioritized in homes and frontlines. | 4 | Conceptualizing primary care as thin hospital / specialist outreach. | 4 | Developing primary care as a full-service preventive, promotive, and curative offering. |
| 5 | Bottle necking of services due to undersupplied skills and expertise | 5 | Augmenting human resources for health with tools & decentralization. |  |  |  |  |
| 6 | Reactive population-level interventions. | 6 | Population-scaled intelligence and proactive strategies. |  |  |  |  |
| 7 | Late diagnoses with complications or undiagnosed conditions due to lack of access and/or knowledge. | 7 | An early screening, prediction, diagnosis system based on surveillance, monitoring ,and adequate knowledge. |  |  |  |  |
| 8 | Using data for reporting. | 8 | Utilizing data and data systems to support caregivers and improve services. |  |  |  |  |
| 9 | Limited or no access of citizens to personal health records and system performance data | 9 | Full and free access and control of personal health records and performance data, available in a language that can be comprehended easily by citizens. |  |  |  |  |
| **Human Resources for Health** | | | |  |  |  |  |
| 1 | Focusing investments in highly specialized HRH | 1 | Building strong team-based care with a focus on family medicine (pride and ownership within the family medicine/general medicine community) |  |  |  |  |
| 2 | Majority of HRH policies surrounding only licensed practitioners | 2 | Politically acknowledging and including all human resources for health personnel within HRH policies/structures, career progression plans, compensation/incentives |  |  |  |  |
| 3 | National approach to allocation, placement, and distribution (top/down approach) | 3 | State-specific (contextualized) approach [distribution, training should take this into account] |  |  |  |  |
| 4 | A public sector focus in HRH training, employment, progression | 4 | A whole of system approach for HRH, measuring and fulfilling need in both public and private sector |  |  |  |  |
| 5 | Didactic provider-patient relationship | 5 | A dynamic patient-provider relationship through empowerment of patients and contextualized care by provider. |  |  |  |  |
| 6 | Static HRH roles and responsibilities | 6 | A life-cycle approach for HRH and building pride and ownership of their work. Building a long-term system based on taking into account career progression |  |  |  |  |
